# Editorial: Animal consciousness: exploring theoretical, methodological and ethical issues

**DOI:** 10.3389/fpsyg.2025.1627261

**Published:** 2025-06-12

**Authors:** Matteo Laurenzi, Antonino Raffone, Günter Ehret

**Affiliations:** ^1^Department of Psychology, Sapienza University of Rome, Rome, Italy; ^2^Institute of Neurobiology, University of Ulm, Ulm, Germany

**Keywords:** animal consciousness, animal sentience, consciousness, multimodal framework, phenomenology, animal cognition, evolution of sentience

## Introduction

Progress in neuroscience, cognitive ethology, and philosophy of mind has driven renewed interest in the study of animal consciousness (Andrews et al., 2024; Ehret and Romand, 2022; Bayne et al., 2024; Grinde, 2024). This Research Topic brings together diverse contributions (empirical, theoretical, and conceptual) that propose new ways to think about the nature and evolution of non-human consciousness.

## Recent perspectives

An evolutionary framework sets the stage for the approaches presented in the following studies. In his first article, Lacalli(a) proposes that consciousness and agency, which are typically seen as co-evolving, may have distinct evolutionary origins. The author suggests that conscious experience may have preceded the emergence of full individual agency, and outlines three functional levels: conscious sensations, consciously conditioned reflexes (CCRs), and deliberate choices (DCs). While DCs involve memory and intention, CCRs describe intermediate behaviors modulated by conscious input without deliberate control.

In his second contribution to this Research Topic, Lacalli(b) explores adaptive functions of consciousness, distinguishing general roles (such as behavioral flexibility) from more specific functions related to memory and motivation. He introduces the speculative concept of “species memory,” by which qualia could encode ancestral experience, allowing consciousness to shift from moment-to-moment regulation to broader integrative functions across evolutionary time.

Kaufmann, in turn, addresses methodological and ethical issues. She contrasts the “markers hypothesis,” which seeks discrete indicators of consciousness, with the “universal consciousness hypothesis,” which assumes the presence of consciousness unless disproven. Arguing that both hypotheses carry risks, the author advocates for a middle path: a dimensional, species-sensitive model that aligns empirical caution with ethical responsibility.

Other articles address the question of whether (and how) consciousness can be studied empirically. Irwin(a) proposes a symbolic framework to profile consciousness across species. Avoiding anthropocentric criteria, he develops a behavioral matrix based on volition, interaction, and self-direction, quantified through measurable variables such as frequency and dynamism. Irwin's(b) second article deepens his analysis through gaze-shift patterns in birds and lizards. Noting marked differences in the frequency of attention redirection, he suggests that even simple behaviors such as eye movement can illuminate how different animals become aware of their environments.

Following in the tradition of human consciousness research, Gutfreund cautions against the over-interpretation of neural and behavioral data. While recognizing recent progress in animal neuroscience, he argues that such data alone cannot resolve the subjective nature of consciousness. Emphasizing the gap between observation and attribution, he calls for epistemic humility, suggesting that consciousness, as a first-person phenomenon, may remain inaccessible to third-person methods. What this means for animal research remains an open discussion.

Woodruff offers a bridging perspective through the case of tonic immobility, a widespread defensive behavior among animals. Its initiation appears to be automatic, but its termination is responsive to environmental context, potentially reflecting rudimentary awareness. Without invoking higher cognition, Woodruff cautiously interprets this pattern as consistent with low-level forms of anoetic consciousness.

Feinberg proposes a broader biological and evolutionary account of sentience (phenomenal, primary consciousness). His theory of Neurobiological Emergentism holds that subjective experience arises through increasing neurobiological complexity and hierarchical integration. Sentience, in this view, is an emergent property of evolved nervous systems. In contrast to theories rooted in biopsychicism such as Integrated Information Theory, Feinberg reframes the explanatory gap as an “experiential gap” i.e., a natural and expected outcome of biological emergence. Sentience is both personal and physical: first-person in character, yet fully explicable within the framework of evolutionary neuroscience.

A final group of contributions expands the conceptual framework of animal consciousness. Laurenzi et al. propose that the study of consciousness must reckon with the self not as an afterthought, but as a central, multifaceted phenomenon in its own right. Drawing on the Pattern Theory of Self, they argue that self-identity is best understood as a dynamic constellation of bodily, affective, cognitive, and social dimensions. This multidimensional view avoids the binary traps of minimal vs. reflective self-awareness, and resists anthropocentric benchmarks such as mirror self-recognition.

Homberg et al. reflect on the tensions between animal and animal-free research. Drawing on their own experiences, they advocate for an ethic of mutual respect, one that recognizes different methodological commitments not as moral oppositions, but as diverse strategies in the shared pursuit of knowledge. They call for open, context-sensitive dialogue, suggesting that the way we communicate about consciousness in animals affects not only public understanding but also the ethical landscape in which animal research is conducted.

## Conclusion and future directions

This volume marks a clear shift toward animal-centered, pluralistic and graded approaches to the evolution of consciousness reflecting biological diversity and epistemic humility. Rather than focusing on whether animals are conscious at all, the focus is on how consciousness may have evolved to help animals adapt to their ecological niches and on how we should ethically and scientifically treat animals according to their level of consciousness Two shared commitments stand out: a rejection of consciousness as a single trait in favor of a dynamic integration of cognitive, affective, and perceptual processes including their neural backgrounds, and a methodological self-awareness that recognizes the limits of inference and the moral risks of misattribution.

Looking ahead, the field calls for integrative methods that can provide adequate definitions of animal consciousness, including discussion about the plausibility of collective consciousness in animals, refine behavioral and physiological research tools, build cross-species models that are free of anthropocentrism, and embed ethical reflection into research practices. Rather than offering definitive answers, the present essays reflect inquiries that may become important if we are to understand consciousness in its many forms, and in doing so, reconsider our own place in the living world.
